# Hematopoietic Stem Cells in Wound Healing Response

**DOI:** 10.1089/wound.2021.0065

**Published:** 2022-08-16

**Authors:** Norifumi Urao, Jinghua Liu, Kentaro Takahashi, Gayathri Ganesh

**Affiliations:** Department of Pharmacology, State University of New York Upstate Medical University, Syracuse, New York, USA.

**Keywords:** hematopoietic stem cell, myelopoiesis, myeloid progenitor, bone marrow, niche, immune memory

## Abstract

**Significance:**

Emerging evidence has shown a link between the status of hematopoietic stem cells (HSCs) and wound healing responses. Thus, better understanding HSCs will contribute to further advances in wound healing research.

**Recent Advances:**

Myeloid cells such as neutrophils and monocyte-derived macrophages are critical players in the process of wound healing. HSCs actively respond to wound injury and other tissue insults, including infection and produce the effector myeloid cells, and a failing of the HSC response can result in impaired wound healing. Technological advances such as transcriptome at single-cell resolution, epigenetics, three-dimensional imaging, transgenic animals, and animal models, have provided novel concepts of myeloid generation (myelopoiesis) from HSCs, and have revealed cell-intrinsic and -extrinsic mechanisms that can impact HSC functions in the context of health conditions.

**Critical Issues:**

The newer concepts include—the programmed cellular fate at a differentiation stage that is used to be considered as the multilineage, the signaling pathways that can activate HSCs directly and indirectly, the mechanisms that can deteriorate HSCs, the roles and remodeling of the surrounding environment for HSCs and their progenitors (the niche).

**Future Directions:**

The researches on HSCs, which produce blood cells, should contribute to the development of blood biomarkers predicting a risk of chronic wounds, which may transform clinical practice of wound care with precision medicine for patients at high risk of poor healing.

**Figure:**
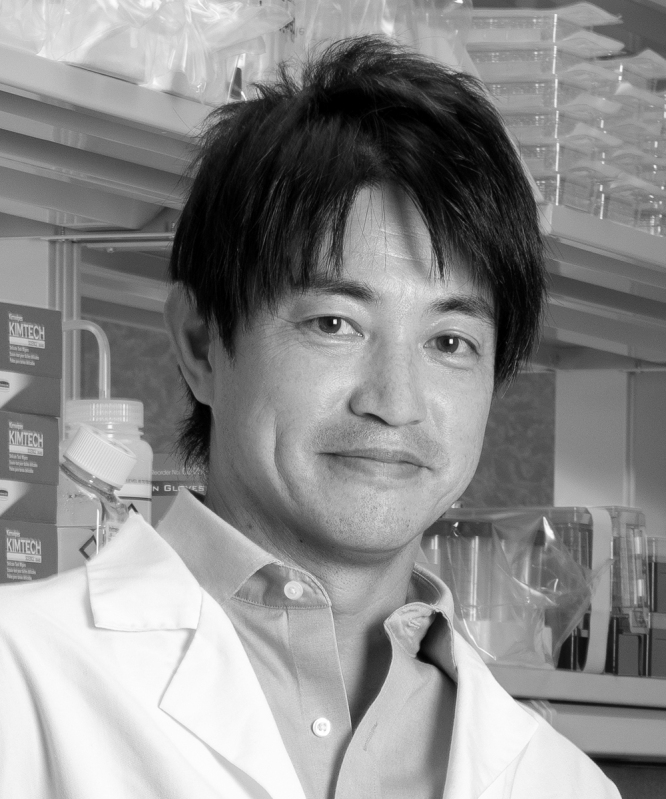
Norifumi Urao, MD, PhD

## Scope and Significance

Emerging evidence has shown a link between the status of hematopoietic stem cells (HSCs) and wound healing responses. The key processes of this link are myelopoiesis in bone marrow (BM) and myeloid responses in the wound. We summarize recent advances in the role of HSCs in myelopoiesis, healing responses, and disease progression. We also highlight mechanisms of dysfunction of HSCs in conditions associated with nonhealing wounds. These conditions, including obesity, diabetes, hypercholesterolemia, atherosclerosis, phycological stress, and aging, not only develop HSC dysfunction but also are comorbidities of chronic wounds. Thus, a better understanding of HSCs will contribute to further advances in wound healing research. This review should be considered a narrative rather than a systematic review.

## Translational Relevance

A large majority of evidence presented in these review articles are from animal models, especially mouse models, because of the nature of HSCs, which is difficult to access and is almost impossible to study their functions in a whole body in humans. However, carefully designed studies show clear similarities of HSC functions between humans and mice. Thus, elucidating the mechanisms of how HSCs contribute to healing and of how dysregulation of HSCs leads to nonhealing, will allow us to translate into useful biomarkers and novel therapy for better wound healing.

## Clinical Relevance

Better wound care requires risk stratification of poor healing. The researches on HSCs, which produces blood cells in response to the wound, will give us opportunities to establish biomarkers in blood cells and serum and to transform clinical practice of wound care with precision medicine for patients with high nonhealing risk.

## Overview

HSCs and hematopoietic progenitor cells, collectively called hematopoietic stem progenitor cells (HSPCs) maintain the homeostatic output of blood cells throughout the lifespan, and their deteriorations cause hematologic disorders, including anemia, bleeding or clotting disorders, and blood cancers. At the same time, many diseases and conditions that accompany damaged tissues in the organs other than the hematopoietic system exhibit hematopoietic abnormality.

Hematopoiesis occurs on-demand in a wound injury involving blood loss and acute inflammation. In blood loss, erythroid progenitor cells, as well as their upstream HSPCs, become activated to generate red blood cells as well as white blood cells or leukocytes. In acute inflammation, generating leukocytes through hematopoiesis is crucial to restoring tissue integrity. The demand for myeloid cells (especially neutrophils and monocytes) increases as an extent of tissue injury gets larger. Myelopoiesis rates are adjusted accordingly to meet the additional need for innate immune cells and to replenish depleted reserves, at the expense of the erythroid and lymphoid cell generation.

Compared with lymphocytes, myeloid cells are more relying on their generation through hematopoiesis (myelopoiesis) because they have a relatively short lifespan and their number reflects innate immune responses. Myeloid cells such as neutrophils, monocytes/macrophages, and dendritic cells are major players in wound healing. Studying injury-induced myelopoiesis should provide a better understanding of myeloid-mediated healing responses.

Hematopoiesis, especially enhanced myelopoiesis, is suggested to contribute to chronic inflammation, which is often accompanied by leukocytosis. The conditions with chronic inflammation increase the risk for nonhealing wounds or chronic wounds, which represent a significant health problem in the United States with millions of patients afflicted and the associated treatment costing billions of dollars per year.^1,2^ Such conditions, including obesity and/or diabetes, autoimmune, atherosclerosis, pre-existing cancer, and infections are associated with dysregulated myelopoiesis and myeloid system, which are often seen as leukocytosis.

In humans, leukocytosis is an established risk factor for cardiovascular diseases such as myocardial infarction and ischemic stroke,^3–6^ and acute osteomyelitis of the foot.^7^ Preoperative asymptomatic leukocytosis is associated with adverse postoperative outcomes after cardiac surgery and is an independent predictor of infection-related postoperative complications.^8^ Not only are the levels increased, but these innate immune cells, such as monocytes/macrophages and neutrophils, are also persistently activated.

Despite their increased levels and proinflammatory activation, in some cases of disease-associated leukocytosis, there is a lack of sufficient acute response to infectious and noninfectious insults, suggesting that dysregulated myeloid systems are beyond overly simplified to two major types of monocytes characterized in mice and humans.^9^ As a vast majority of the people who have a prolonged open wound usually also have other major health conditions, dysregulated myelopoiesis in both quantity and quality may be a key pathological feature in comorbidities of nonhealing wounds.

## Discussion

### Myelopoiesis during wound healing

#### Myelopoiesis from HSCs

Myelopoiesis is a complex process consisting of proliferation, differentiation, and migration. The BM is a major reservoir of differentiated cells, and differentiated myeloid cells are mobilized from BM to the blood circulation. The myelopoiesis as well as the mobilization of differentiated cells are governed by intricate mechanisms linked with cells and factors in the microenvironment for HSCs or the niche. Not only fully differentiated cells, but progenies from HSCs that include multipotent progenitors (MPPs) and myeloid progenitors (MyPs) in multiple stages are also likely regulated by their specific niche, which eventually contributes to inflammation and myeloid responses.

**Figure 1.**
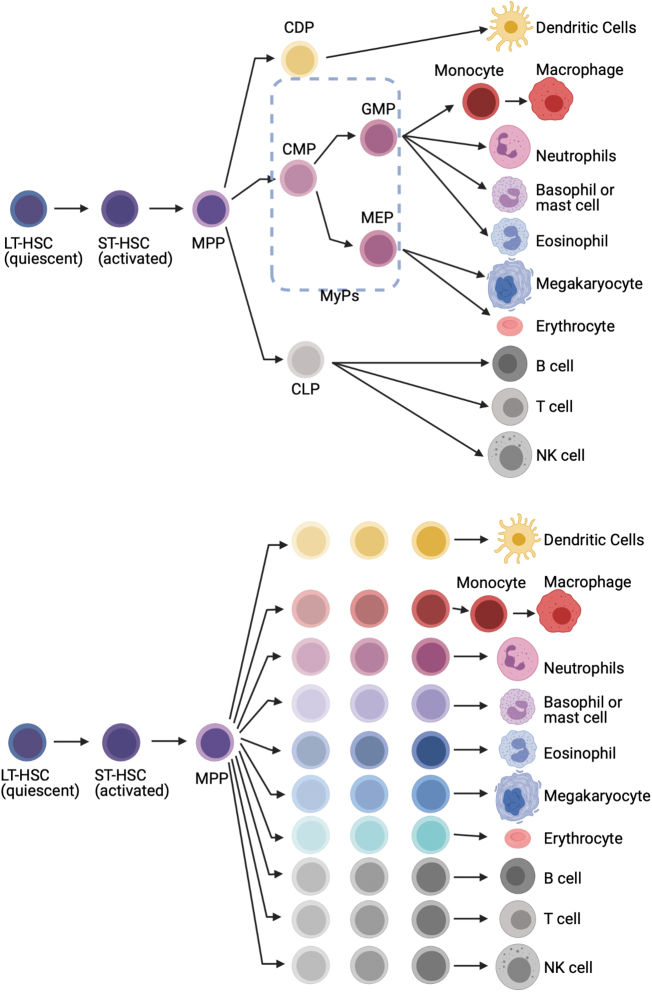
Models of hierarchical differentiation of HSC. **(a)** Classical “step-wise” model of hematopoiesis. HSCs differentiate into terminally differentiated cell types through discrete hierarchically organized progenitor populations. In this model, HSCs are thought to be homogeneous and multipotent to be differentiated to any cell types. **(b)** “Continuous” differentiation model of hematopoiesis. Recent advances in single-cell RNA sequencing revealed that HSCs are a more heterogeneous population consisting of cells with lineage-specific fates and HSCs differentiate into specific cell types without any major transition through the multi- and bipotent progenitors as described in “step-wise” model. HSC, hematopoietic stem cell.

Multi- or oligopotent progenitors were defined by clonal colony-forming assays with lineage-specific hematopoietic cytokines, and surface marker profiling by flow cytometry. Granulocytes (including neutrophils), monocytes, and dendritic cells are thought to be produced by granulocyte monocyte progenitors and monocyte-dendritic cell progenitors, which are themselves derived from common myeloid progenitors (CMPs). However, newer cell fate-tracking approaches demonstrate a prominent role for MPPs in maintaining myeloid production in the steady state.^10–12^ Detailed functional studies of the MPP compartment have identified three distinct MPP subsets (MPP2, MPP3, and MPP4). Increased generation of myeloid-biased MPP2 and MPP3 from HSCs together with myeloid reprogramming of lymphoid-biased MPP4 is shown as key first steps of regenerative myelopoiesis.^13^ This “stepwise” model of hematopoiesis does not cover whole aspect of myelopoiesis. The “continuum” model of hematopoiesis captures heterogeneity of progenitors in multiple stages (Fig. 1).

#### Myeloid responses in the phases of wound healing

Wound healing of injured tissues is characterized by three overlapping phases—inflammatory, proliferative, and resolution. These phases should progress with timely transitions for the restoration of tissue functions. Nonhealing or poor healing with compromised tissue functions is associated with a lack of phase transitions and with persistent inflammation. Thus, initiating phase transitions in nonhealing wounds may lead to better healing. The proper phase transitions of wound healing require orchestrated myeloid responses that accompany inflammatory and healing signals, whereas nonhealing wounds are associated with proinflammatory myeloid accumulation and chronic inflammation.^14^

Neutrophils and proinflammatory monocytes/macrophages are accumulated early after the onset of injury in the inflammatory phase. These proinflammatory myeloid cells are gradually replaced by prohealing-type macrophages, creating a biphasic myeloid response in normal healing.^15,16^ Past studies identified key molecular pathways for the biphasic response in the injured tissue locally, such as mediators released from proinflammatory myeloid cells (*i.e.,* resolvins, cytokines, etc.) and macrophage uptake of apoptotic cells (efferocytosis).^17,18^ However, in chronic inflammation with nonhealing, studies have shown continuous replenishment of proinflammatory myeloid cells.^19–22^ Thus, understanding the mechanisms of the proinflammatory myeloid cell replenishment should help to develop an intervention of nonhealing wounds.

During normal healing, the inflammatory phase is followed by proliferation and resolution phases of wound healing. Timely phase transition from the inflammatory phase involves phenotypic switch of myeloid cells, which have often been described in the overly simplified M1 versus M2 polarization of macrophages.^15^ Similar proinflammatory versus anti-inflammatory polarization has been proposed in neutrophils (N1 vs. N2).^23^ In the proliferation/resolution phase, anti-inflammatory myeloid cells play critical roles in tissue normalization through vessel maturation and matrix remodeling. At least in macrophages, which is known to be highly plastic and responsive to environmental changes, the phenotypic switch is partly mediated through the mechanism involving signals from cellular environment, intracellular signaling, and transcriptional reprogramming. However, the generation and recruitment of myeloid subsets should contribute to overall phenotypic switch in the wounds as nonclassical Ly6C^lo^ monocytes give rise to only CD206^+^ wound healing macrophages.^24^

#### Wound-induced myelopoiesis of HSPCs

Myelopoiesis, the generation of myeloid cells, occurs mainly in the BM. To maintain levels of myeloid cells in the blood and peripheral tissues in a normal steady state, myelopoiesis is regulated by the feedback mechanisms that can turn on or off the myelopoietic drive. The early hematopoiesis or the activation of HSPCs is required for proper healing and resolution of inflammation such as in mouse models of ischemic organ injury and sepsis.^25–27^

For example, ischemic stroke activates HSCs through increased sympathetic tone, leading to a myeloid bias of hematopoiesis and higher BM output of inflammatory Ly6C^high^ monocytes and neutrophils.^28^ In myocardial infarction, a CCR2^+^CD150^+^CD48^+^LSK (Lineage^−^Sca1^+^cKit^+^) hematopoietic subset is the most upstream contributor to emergency myelopoiesis after ischemic organ injury.^25^ In hindlimb ischemia, LSK cells and MyP are quickly activated but restore to steady-state levels several days after the tissue injury.^29^ Moreover, injection of skeletal muscle homogenate as damage-associated molecular patterns (DAMPs) are sufficient to increase cell cycling in LSK cells.^29^ In dorsal skin punch biopsies, HSPC activation was observed as increased myeloid-committed MPPs after skin wounds.^30^ Newly generated and mobilized myeloid cells manifest acute inflammation, whereas chronically generated myeloid cells lead to chronic inflammation. Overall, appropriately activated and perhaps a timely limitation of activated HSPCs are critical for wound healing responses.

### Mechanisms of HSPC activation

#### Signals for acute activation of HSPCs

**Figure 2.**
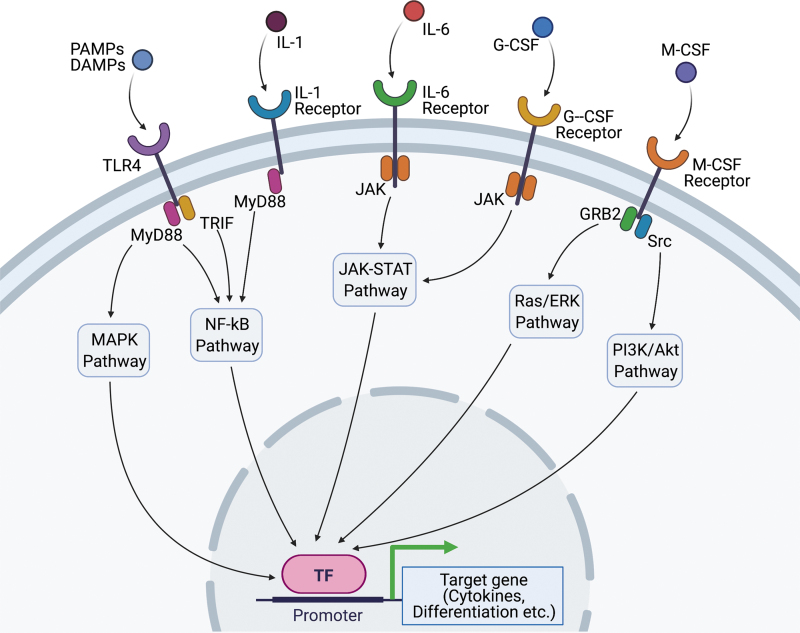
The acute signals of HSPCs in response to external stimulations such as infections or wound damage/tissue ischemia. Circulating cytokines, including G-CSF, M-CSF, IL-1, and IL-6 are increased in the acute phase of infectious diseases and tissue ischemia. G-CSF binds to G-CSF receptor and induces JAK/STAT pathway resulting in upregulation of genes related to granulopoiesis, such as C/EBPβ. M-CSF can also activate myeloid differentiation by inducing PU.1 activity, a key regulator of myeloid development, through Src-PI3K pathway. IL-1 and IL-6 regulate myelopoiesis by triggering a broad range of signaling pathways, including NF-κB and JAK-STAT pathway. PAMPs and DAMPs bind to TLR4 and lead to the activation of both MyD88-dependent pathway and TRIF-dependent pathway, causing emergency granulopoiesis. DAMPs, damage-associated molecular patterns; G-CSF, granulocyte colony-stimulating factor; HSPCs, hematopoietic stem progenitor cells; IL, interleukin; JAK, Janus kinases; M-CSF, macrophage colony-stimulating factor; NF-κB, nuclear factor kappa-light-chain-enhancer of activated B cells; PAMPs, pathogen-associated molecular patterns; PI3K, phosphoinositide 3-kinase; Src, proto-oncogene tyrosine-protein kinase Src; STAT, signal transducer and activator of transcription protein; TLR4, toll-like receptor 4; TRIF, TIR-domain-containing adapter-inducing interferon-β.

HSPCs can be responsive to acute inflammatory stimuli induced by tissue injury or wounding. This HSPC's response is known as emergency myelopoiesis or demand-responsive myelopoiesis, typically described in acute infection and sepsis. The stimuli that directly or indirectly activate HSPCs include DAMPs or pathogen-associated molecular patterns (PAMPs), cytokines, and growth factors, promoting transcriptional changes to drive immune responses in hematopoietic cells from BM (Fig. 2).^31^ Most evidence support that HSPCs sense inflammation or tissue damage indirectly through mature hematopoietic cells and nonhematopoietic tissues, and subsequent changes in factors act on HSPC-expressed receptors, inducing proliferation, differentiation, and migration.

Myelopoietic colony-stimulating factor (CSF) and cytokines—granulocyte-CSF (G-CSF), macrophage-CSF (M-CSF), and granulocyte-macrophage-CSF (GM-CSF), interleukin (IL)-1, IL-3, IL-6, and Flt3 ligand—have prominent roles in acute activation of HSPCs. Those cytokines can be increased in the blood circulation in infection or acute inflammation, whereas the sources of the cytokines could be various from a lesion in the local tissue to niche cells in the BM.

G-CSF serves as a major driver of granulopoiesis under both steady-state conditions and emergency conditions, such as infection, by regulating the expression of myeloid lineage-specific transcription factors and of receptors for myeloid lineage-specific growth factors.^32^ G-CSF induces proliferative and lineage-specific signals on HSPCs.^33–35^ BM niche (endothelial cells, mesenchymal stem cells [MSCs]) also contributes to emergency granulopoiesis through G-CSF.^36,37^ G-CSF-induced activation of its receptor, G-CSFR, leads to signaling through the transcription factor STAT3, which directly stimulates expression of the master transcriptional regulator of emergency granulopoiesis, C/EBPβ.^34,38^ Single-cell RNA sequencing showed G-CSF disrupts quiescent HSC signature before expanding MyP populations.^39^ Further analysis using CRISP-seq indicates Cebpα regulates entry into myeloid fates of HSPCs as priming under G-CSF stimulation.^39^

M-CSF also contributes to emergency myelopoiesis. M-CSF acts directly on HSPCs and induces their differentiation into monocytes.^40^ M-CSF can instruct myeloid lineage identity in single HSCs, independently of HSC survival or proliferation, by inducing a PU.1-dependent molecular signature.^41^

IL-1 is a central mediator in response to infections or sterile insults. IL-1 binds a broadly expressed surface receptor (IL-1R) and triggers a wide range of signaling pathways, including NF-κB, p38 MAPK, JNK, and AP-1.^42^ IL-1 acts directly on HSPCs *in vitro*, promoting their proliferation and myeloid differentiation through activation of the transcription factor PU.1, which is a key regulator of myeloid development.^43^ Injection of IL-1β to naive mice is sufficient to induce HSPC activation and myelopoiesis.^43,44^ IL-1β, through increased expression of IL-1R on HSPCs, is a crucial mediator that promotes HSPC expansion and granulopoiesis in postmyocardial infarction.^44^ However, skin wound-induced MPP expansion and blood myeloid profiles are not affected in mice lacking IL-1R, suggesting that other signals are sufficient to induce the HSPC activation in mouse skin wounds.^45^

Tumor-necrosis factor (TNF) has a complex role in hematopoiesis. Based on the analysis on TNF receptor knockout mice, the role for TNF in the survival of HSPCs is positive^46^ or negative.^47^ TNF directly upregulates PU.1 *in vitro* and myeloid generation from HSCs, at the same time TNF promotes apoptosis of MyPs and prevents HSC necroptosis, and induces T cell suppression activity.^48^ In the scenario of BM transplantation, TNF derived from granulocytes acts on BM endothelial cells promoting vessel and hematopoietic regeneration.^49^ The same study also suggests that TNF acts directly on hematopoietic progenitors since the effect of granulocyte transfer was decreased but not eliminated in mice that were transplanted with hematopoietic cells from TNF receptor-deficient mice.^49^ Thus, TNF acting on hematopoietic progenitors promotes hematopoietic regeneration after BM transplantation by amplifying regeneration signals of other cells such as endothelial cells as well as within hematopoietic progenitor cells in a paracrine manner.

Interferons (IFN) protect against infections. In myocardial infarction, a type of resident macrophage produces type I IFN (IFN-α and IFN-β). When these macrophages uptake cell debris, particularly DNA, type I IFN signaling is stimulated and is detrimental.^50^ IFN-α and RNA virus mimicking polyinosinic:polycytidylic acid quickly (in 16 h) drives the proliferation of dormant HSCs in an IFN-α/-β receptor-dependent manner.^51^ Thus, excessive type I IFN signaling in HSPCs is harmful. IFN-γ secreted by effector cytotoxic T cells stimulates hematopoiesis at the level of early multipotent hematopoietic progenitor cells and induces myeloid differentiation. IFN-γ does not primarily affect HSPCs directly. Instead, it promoted the release of hematopoietic cytokines, including IL-6 from BM MSCs in the HSC niche, which in turn reduced the expression of the transcription factors Runx-1 and Cebpα in early hematopoietic progenitor cells and increased myelopoiesis.^37^ IFN-γ signaling is required for the expansion of HSCs and promotion of myelopoiesis induced by Bacillus Calmette-Guérin (BCG) vaccination, which accesses the BM.^52^

IL-6 is a pleiotropic cytokine playing an important role in the acute immune response. IL-6 regulates the proliferation and differentiation of HSPCs during emergency myelopoiesis. In toll-like receptor (TLR) stimulation, IL-6 has been shown as a signaling hub and a particularly important regulator of myeloid differentiation and HSPC proliferation in a paracrine manner, which mediates rapid myeloid cell recovery during neutropenia.^53^

In acute inflammation and/or infection, TLRs (a type of pattern recognition receptor), which recognize conserved microbial or self-tissue products, such as lipopolysaccharide (LPS), play a central role in innate immunity. TLRs are expressed on a wide variety of effector immune cell types, as well as HSPCs.^54^ As mentioned above, after activation of TLRs, activated HSCs and MPPs produce substantial amounts of cytokines in a manner dependent on the transcription factor NF-κB. Those cytokines, particularly IL-6, can promote myelopoiesis of HSPCs in a paracrine fashion.^53^ Direct activation of TLR4 on HSPCs stimulated their proliferation but may have some detrimental effects on their function due to the proliferative stress in HSCs after ligation of TLR4.^55,56^ TLR4 recruits members of a set of toll/IL-1 receptor domain-containing adaptors, such as MyD88 and TIR domain-containing adapter-inducing interferon-β (TRIF). MyD88-dependent pathway activates NF-κB and mitogen-activated protein kinase (MAPK) for induction of inflammatory cytokine genes, while TRIF-dependent pathway promotes an alternative pathway leading to the activation of IRF3, NF-κB, and MAPKs for induction of type I IFN and inflammatory cytokine genes.^57^ The TRIF-dependent pathway also mediates the expansion and functional exhaustion of HSPCs in sepsis.^55,56^

In addition to the direct effect of TLR agonists on HSPCs, the TLR4-MyD88 pathway mediates LPS-related emergency granulopoiesis through a paracrine pathway, which requires the production of G-CSF by endothelial cells. In infected wounds, PAMPs, a diverse set of microbial molecules that share many different general patterns or structures, play a role. PAMPs, including LPS and β-glucan are recognized by pattern-recognition receptors, such as TLR4 and Dectin-1, respectively.

#### Signals for chronic activation of HSPCs

Activated HSPCs are linked with chronic inflammation.^41,43,58,59^ In conditions with chronic inflammation (*i.e.,* chronic infection, obesity/diabetes, autoimmune diseases, and atherosclerosis),^59–62^ emerging evidence has shown that myelopoiesis from activated HSPCs play an active role in sustaining higher levels of pro-inflammatory myeloid cells.^31,63^ Such activated myelopoiesis from HSPCs is often seen as “myeloid skewing.” Myeloid skewing of HSPCs is observed as increased number or pool size of myeloid (biased) progenitor cells.

**Figure 3.**
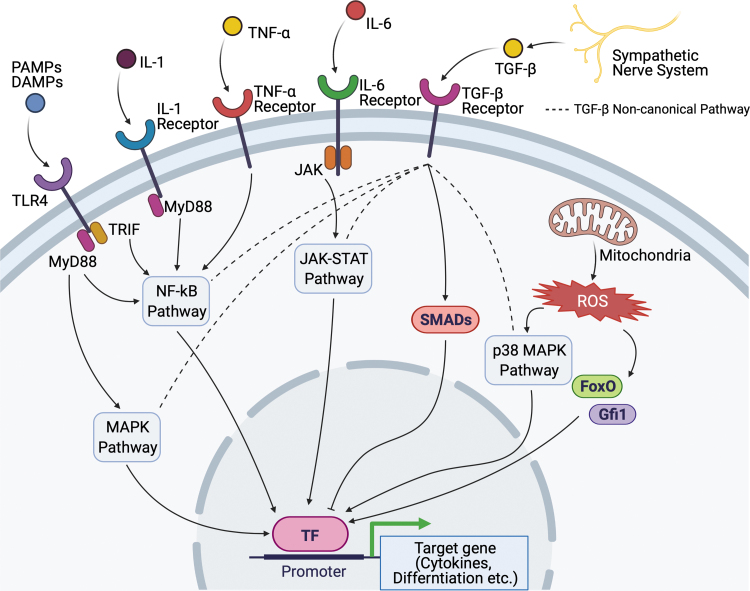
The chronic signals of HSPCs under conditions with chronic inflammation. TLR4, IL-1, and IL-6 are important mediators in chronic signal pathways. They utilize the same downstream pathway to activate myeloid skewing. ROS are produced from mitochondria in reaction to chronic inflammation. ROS dysregulates HSPC function by upregulating the specific transcription factors such as FoxO and Gfi1. p38 MAPK pathway is also activated by ROS resulting in the inhibition of HSPC self-renewal. The sympathetic nerve system plays an essential role in HSPC differentiation as a part of the bone marrow niche. TGF-β is produced from glial cells ensheathing sympathetic nerves and negatively regulates HSPC proliferation. TGF-β noncanonical pathway (*dashed line*) has crosstalk with other signaling pathways to orchestrate HSPC function. FoxO, forkhead box O; Gfi1, growth factor independent 1 transcription repressor; MAPK, mitogen-activated protein kinase; ROS, reactive oxygen species; TGF-β, transforming growth factor-beta.

While MyPs have been characterized based on their surface maker expressions and following the generation of myeloid cells of sorted and transplanted progenitors, more recent studies using transcriptome analysis in single cells and *in vivo* lineage tracing have provided a more complex view of myeloid skewing. For example, CMPs had been defined as the progenitors that have the capability of differentiating into granulocytes, monocytes, dendritic cells, erythroid, and megakaryocytes.^64^ In the steady state alone, single-cell transcriptomic analysis of CMPs in BM revealed early transcriptional priming toward seven different fates.^65^ Moreover, in a model of emergency myelopoiesis, clonal expansion and activation of the “primed” HSPCs, rather than instructing HSPCs with other fates, is observed, at least, in dendritic cell development.^66^ We summarize signals that can cause HSPC activation under conditions with chronic inflammation (Fig. 3).

TLR signaling plays an important role in the regulation of HSPCs in chronic disease conditions. Chronic treatment with a low dose of LPS results in the expansion of HSPCs and their myeloid skewing in a cell-intrinsic, TLR4-dependent manner, as shown in serial transplantation experiments and BM chimera experiments with cells from TLR4-deficient mice and TLR4-sufficient mice.^67,68^ While direct TLR4 activation in HSCs might be beneficial for controlling systemic infection, prolonged TLR4 signaling has detrimental effects and may contribute to inflammation-associated HSPC dysfunction.^56^

IL-1β interacted with the IL-1 receptor on BM MyPs to stimulate the production of monocytes and neutrophils in obesity that upregulates IL-1β secretion from adipose tissue macrophages.^69^ Similarly, in aged mice, IL-1β, together with IL-6 and IL-1α, is increased in BM extracellular space.^70^ Chronic administration of IL-1β diminishes the self-renewal capacity of HSPCs.^43^ IL-1β secretion from BM macrophages, which is increased in aged mice, promotes myeloid-biased CD41^+^ HSCs.^71^ IL-1β and GM-CSF signaling in HSPCs explain increased myelopoiesis of β-glucan-induced trained immunity in mice.^72^

IFN-α drives the proliferation of dormant HSCs and impairs the self-renewal potential of HSCs in a chronic setting.^51^ Type I IFNs impaired hematopoiesis during infection by both limiting HSC/HSPC proliferation and increasing HSPC death.^73^ Infection with *Mycobacterium avium* triggers the proliferation of HSCs in an IFN-γ-dependent manner.^58^ Moreover, IFN-γ is responsible for the attrition of HSCs due to their differentiation into myeloid cells and loss of their self-renewal capacity during chronic mycobacterial infection in mice,^74^ suggesting that prolonged stimulation of HSCs induces their terminal differentiation that causes pancytopenia in chronic inflammation.

TNF-α in chronic stimulation promotes HSC survival and myeloid differentiation by activating a strong and specific p65-NF-κB-dependent gene program that primarily prevents necroptosis, and poises HSCs for myelopoiesis.^48^ TNF-α production from HSPCs is increased in hypercholesterolemia.^75^

Transforming growth factor-beta (TGF-β) has been shown as a niche factor that maintains HSC quiescence and inhibit its expansion^76–79^ partly through inhibiting lipid raft clustering that can be caused by activating cytokines,^80^ whereas myeloid-biased progenitors may be activated by TGF-β.^81^ Diet-induced obesity can disrupt the TGF-β receptor within lipid rafts along with impaired SMAD2/3-dependent TGF-β signaling.^82^ In addition to its canonical pathway through SMADs, TGF-β noncanonical pathway potentially has crosstalk with MAPK pathway, NF-κB pathway, and JAK-STAT pathway, which are the downstream of TLR4, IL-1/6 receptors, and TNF-α receptor.^83^ Endoglin, a type III receptor for the TGF-β superfamily, positively modulates TGF-β signaling to ensure maintenance of HSC quiescence.^84^ In the steady state, megakaryocytes^78^ and Schwann cells of the nerve^79^ have been shown as significant source of TGF-β in the niche.

Oxidative stress is involved in several chronic pathological processes such as obesity,^85^ type 2 diabetes,^85,86^ atherosclerosis, and aging. Reactive oxygen species (ROS), which is the major source of oxidative stress, act through p38 MAPK to limit the lifespan of HSPCs.^87^ Increased levels of ROS during aging induces myeloid lineage skewing and defective long-term repopulation activity, as has been demonstrated in the case of FoxO (forkhead box O) transcription factors' depletion in mice.^88,89^ FoxO is one of the central regulators of the HSC stress response by controlling self-renewal, proliferation, and survival.^90^ Obesity is also associated with an increased level of intracellular ROS.^91^ ROS upregulates Gfi1, which controls HSC quiescence, in obese mice resulting in long-lasting dysregulated HSPC function.^85^

Conditions with chronic inflammation involve mechanisms that sustain hyperactivation and compromised function of HSPCs. These are explained by cell-intrinsic mechanisms—epigenetic (and/or genetic) reprogramming, and cell-extrinsic mechanisms—remodeling of the microenvironment for HSPCs. We will discuss this in more detail in the following sections.

#### Epigenetic programming of HSPCs

**Figure 4.**
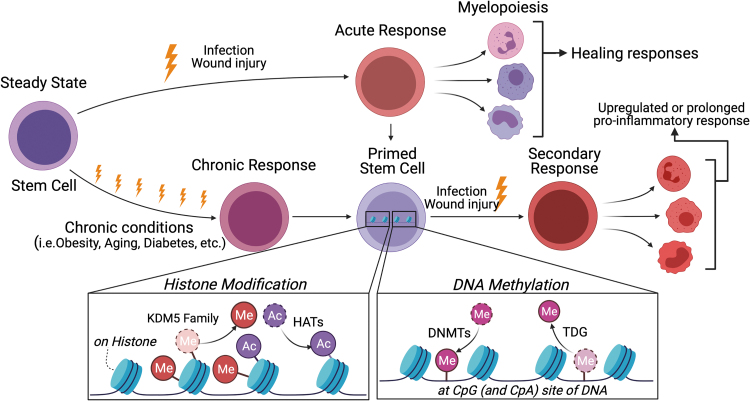
Epigenetic reprogramming of HSPCs that potentially dysregulate wound healing. Innate immune memory is provoked by both acute and chronic stimuli. Through these stimuli, HSPCs are “primed” by epigenetic reprogramming such as histone modification and DNA methylation. Histone methylation and acetylation are regulated by KDM family and HATs, respectively. DNMTs mediate DNA methylation by transferring a methyl group to DNA, while TDG plays essential roles in the demethylation process. When “primed” HSPCs are exposed to secondary stimuli, increased or tolerated response is induced, leading to dysregulated myelopoiesis and cytokine expression. DNMTs, DNA methyltransferases; HATs, histone acetyltransferases; KDM, histone lysine methyltransferase; TDG, thymine DNA glycosylase.

Exposure to cytokines or microbial products trains HSPCs by reshaping the epigenetic landscape. Trained HSPCs show enhanced myelopoiesis against the subsequent infectious events (known as trained immunity).^92^ Chronic disease conditions, such as obesity,^93^ diabetes,^94^ arthritis, atherosclerosis, and aging,^95^ also induce epigenetic reprogramming in HSPCs to regulate their proliferation and differentiation to MyP cells. Epigenetic programming includes chromatin remodeling such as histone modifications, DNA methylation, and noncoding RNA such as micro-RNA and long noncoding RNA (lncRNA) (Fig. 4).

Histone 3 lysine 27 acetylation (H3K27ac) and histone 3 lysine 4 trimethylation (H3K4me3) are key epigenetic marks in reprogramming by immune responses. H3K27ac and H3K4me3 are enriched at distal enhancers and the promoters of stimulated genes, respectively. Emerging evidence demonstrated that pathological conditions cause histone modification in monocytes or macrophages.^96,97^ β-Glucan induces upregulation of H3K27ac and H3K4me3 enrichment both at enhancer and promoter regions, resulting in dysregulation of transcription factors associated with inflammation.^96^ Similarly, BCG-educated HSPCs modified H3K27ac and H3K4me3 enrichment in macrophages that provide significantly better protection against virulent Mycobacterium *tuberculosis* infection than naive macrophages.^52^

Several pathways have been proposed to explain the mechanism underlying histone modification in response to external stimuli. Trained immunity has been shown to associate with metabolic changes induced by external stimuli.^98^ Histone modification is provoked through several metabolites from glycolysis and the tricarboxylic acid cycles. β-Glucan upregulated H3K4me3 enrichment by inhibition of histone demethylase KDM5 (also known as Jarid) due to accumulation of fumarate, which is a key metabolite in mediating β-glucan-induced trained immunity in monocytes.^99^ Diet-induced obesity increases H3K4me3 at KDM6B (also known as Jmjd3), H3K27 demethylase in MyP, which in turn leads to an enhanced inflammatory phenotype of macrophages.^100^ Sun *et al.* showed direct evidence of upregulated H3K4me3 enrichment in aged HSPCs.^101^ These studies showed histone modifications at targeted gene loci, however, the full landscape of histone modifications in HSPCs remains to be understood. The development of a methodology to analyze the epigenetic landscape in small cell numbers will overcome the difficulties in obtaining sufficient cell yield for chromatin immunoprecipitation with sequencing.

DNA methylation is one of the best-studied epigenetic systems in mammals. DNA methylation usually occurs at CpG islands, which are found at the majority of promoters.^102^ The presence of DNA methylation at CpG islands is usually associated with long-term, stable gene repression. DNA methylation is predominantly established by DNA methyltransferases (DNMTs) and oxidized by ten-eleven translocation (TET) enzymes. Under physiological conditions, DNA methylation undergoes extensive changes during HSPCs differentiation while maintaining HSPC homeostasis.^103^ Mutations in DNMTs or TET enzymes cause abnormal DNA methylation leading to deficiency of HSPC differentiation. Thus, mutations in DNMTs or TET enzymes are involved in the occurrence of leukemia.^104,105^

Deficiency of TET2 accelerates atherosclerosis development due to clonal expansion of TET2-deficient HSCs resulting in upregulated chemokine and cytokine levels.^106^ Alteration of DNA methylation occurs in genomic regions associated with hematopoietic lineage potential during chronic pathological conditions such as aging and obesity.^101,107,108^ Altered DNA methylation reinforces HSPC self-renewal and diminishes differentiation, resulting in phenotypic HSPC aging behavior.^101^ Dietary fat-induced hypercholesterolemia in Ldlr^−/−^ mice showed altered BM methylation patterns at the promoter regions of PU.1 and Irf8 genes, important for monocyte and macrophage hematopoiesis.^108^

Noncoding RNAs involve cellular reprogramming. MicroRNAs (miRs) are small noncoding RNAs containing about 22 nucleotides that mediate gene silencing by interacting with target sites in the 3′ untranslated region of mRNAs. Importantly, miRs are highly conserved across species. Multiple miRs have been found to have specific effects on the behavior of HSPCs.^109^ While miR-181, miR-223, and miR-142 induce differentiation of HSPCs,^109^ miR-132 keeps HSPCs in a more quiescent state by inhibiting the expression of FOXO3, which is one of the aging-related transcription factors.^110^

lncRNAs are the different types of noncoding RNA, which can interfere with gene expression and post-transcriptional signaling at various levels.^111^ lncRNAs can directly interact with DNA to recruit epigenetic modulators, act as a decoy for transcription factors, control splicing, or interact with proteins to control protein complex formation.^111,112^ Note that lncRNAs lack conservation across species. lncRNAs have been shown to control self-renewal and lineage commitment of HSPCs under steady-state conditions.^113^ Some lncRNAs such as MALAT1 are involved in the pathophysiology of atherosclerosis by upregulating hematopoiesis in the obese mouse model.^114^

An important aspect of HSPC regulation by noncoding RNAs is that they can be transferred to HSPCs from other surrounding cells that originated them (typically a form of extracellular vesicles [EVs]). Indeed, at least in *in vitro*, mRNAs and miRs can be transferred from stromal cell-derived EVs to LSK-HSPCs that uptake the EVs.^115^ Thus, noncoding RNAs are promising targets as novel biomarkers and therapeutics, although further investigation will be required to understand the more detailed functions and mechanisms of noncoding RNAs in HSPCs.

### HSPC niche in the BM

#### Overview of the niche

HSPCs are maintained in a specific local environment in the BM or “niche” that regulates the quiescence, proliferation, and differentiation of these cells.^116^ Historically, the niche has been characterized in the maintenance of HSCs that is responsible for hematopoiesis throughout life.^117^ Thus, disruption of the niche results in either or a combination of exhaustion of stem cells and/or dysregulated generation of a particular lineage of blood cells.

**Figure 5.**
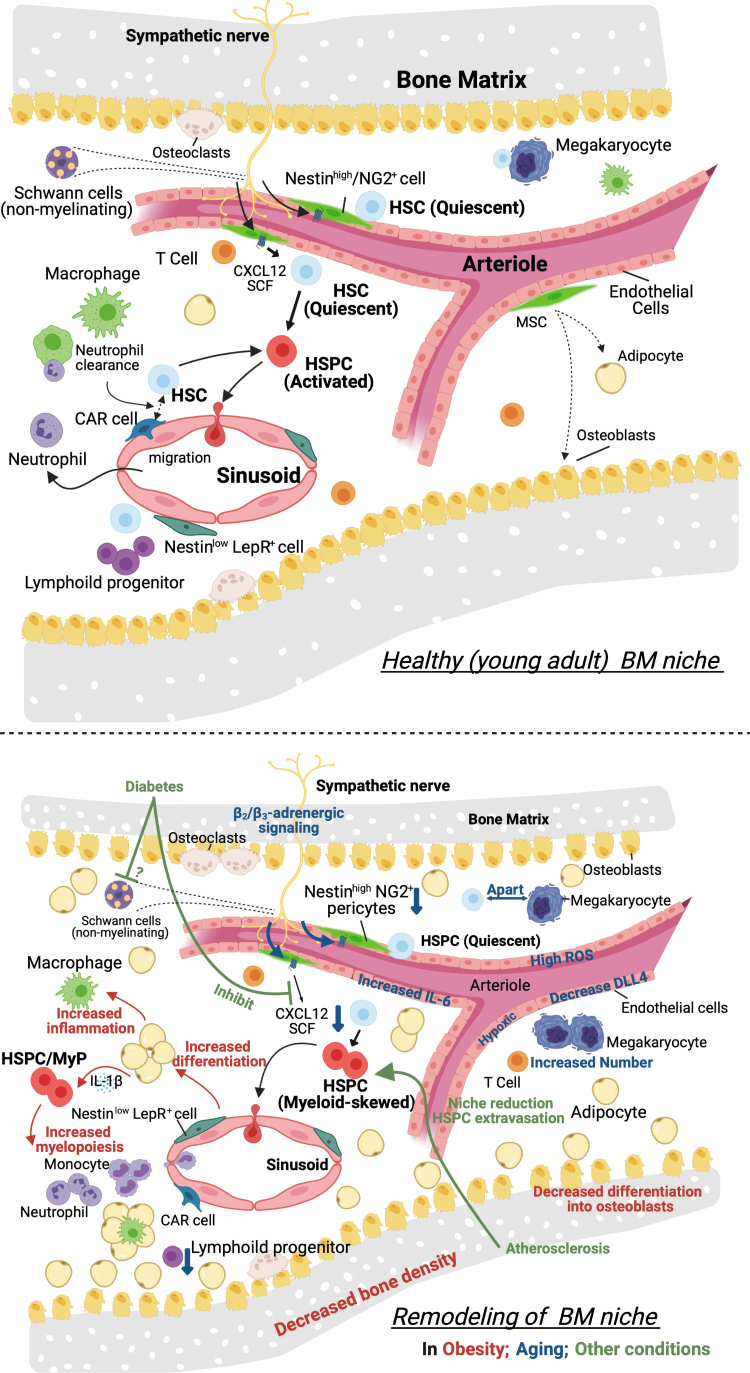
Bone marrow HSPC niche and its remodeling.

In contrast to the HSC niche, the environment for progenitor cells is less characterized. This is because of the methodological limitations of how to identify and localize the niche—a lack of exclusive markers (overlapping of niche factors with HSCs niche). To study hematopoiesis in inflammatory responses, identification of extrinsic signals promoting maintenance and differentiation of hematopoietic progenitor cells is important but is not well understood. Moreover, innate immune memory, which results in either a chronic hyperinflammatory state or a persistent state of immunological tolerance, may be accompanied by alteration or adaptation of the BM niche. We will summarize evidence of niche regulations that are potentially relevant to myeloid responses during wound healing and also niche dysregulations that could be related to the conditions with impaired wound healing (Fig. 5).

#### Cellular components of the niche

HSPCs in the BM are surrounded and their functions are directly or indirectly regulated by other cells, such as osteoblast,^118,119^ osteoclast,^120–122^ stromal cells,^118,123,124^ macrophages,^35,125^ endothelial cells, adipocytes,^79^ megakaryocytes,^78,126^ T cells, nonmyelinating Schwann cells,^79^ and neurons^123,127,128^, consisting of the HSPC niche. HSCs are defined as cell populations that are serially transplantable through multiple recipients. The majority of these long-term HSCs express signaling lymphocytic activation molecule (SLAM or CD150) and localize adjacent to sinusoid vessels (within five cell diameters of a sinusoid).^129^ This perivascular niche includes endothelial cells, and perivascular stromal cells or MSCs.

In acute inflammation or induction of wounding, those niche cells sense signals from damaged tissue in similar ways that HSPCs sense the signals—directly through receptors expressed on the niche cells or indirectly sensing signals amplified by other niche cells. The stimulated niche cells can be the cells (typically immune cells) that home to the niche after traveling in the circulation.

In conditions with chronic inflammation, changes in the composition of the niche are induced and might contribute to perpetuating the conditions through enhanced myelopoiesis. For example, single-cell RNA sequence that aimed to show a transcriptional landscape of mouse BM populations^130^ revealed a considerable transcriptional remodeling of niche elements under stress conditions mimicking chemotherapy (ablation of rapidly dividing hematopoietic progenitor cells and the induction of proliferation and differentiation of otherwise quiescent HSCs). This myeloablation affects sinusoidal vessels^33^ as well as stromal cells and causes an increase in the frequency of BM adipocytes.^131^

#### Vascular and perivascular cells in the niche

Since vascular niches supporting long-term HSCs were identified,^129^ perivascular stromal cells and vascular endothelial cells have been extensively studied. Perivascular stromal populations are marked by various genetic labeling methods, such as the CXCL12-abundant reticular (CAR) cells, Nes-GFP^+^, Scf-GFP^+^, Ng2-Cre^+^, Lepr-Cre^+^, or Prx1-Cre^+^ cells. These are largely overlapping mesenchymal cell populations in the adult BM. They are the main contributors producing key soluble factors that maintain HSCs, the niche factors, such as CXCL12 and stem cell factor (SCF).

Almost all HSCs near the sinusoidal endothelium are in contact with a small population of reticular cells expressing high amounts of CXCL12, called CAR cells.^132^ Nestin is expressed in a subset of BM MSCs that are involved in HSC maintenance and trafficking, and perivascular nestin-GFP bright cells define a quiescent HSC niche.^123^ Prx1 marks mesenchymal progenitor cells and Prx1-Cre targets almost all CAR cells.^133^ Stromal cells expressing leptin receptor (LEPR) is a source of SCF.^124^ Like other MSCs, LEPR^+^ stromal cells can differentiate into adipocytes or osteoblasts. NG2^+^ arteriole perivascular cells harbor lymphoid-biased HSCs^134^ as well as quiescent HSCs.^135^ Depletion of NG2^+^ arteriolar niche cells selectively depletes vWF^−^ lymphoid-biased HSCs.^134^ Osteogenic growth factor, osteolectin, can distinguish between osteogenic LEPR^+^ cells and adipogenic LEPR^+^ cells. Osteolectin^+^LEPR^+^ stromal cells are periarteriolar localized, mechanosensitive, and maintain lymphoid progenitors.^136^ Osteolectin^−^LEPR^+^ stromal cells are perisinusoid localized and maintain HSCs.^136^ Cycling HSCs or MPPs tend to be localized with perisinusoid (osteolectin^−^) LEPR^+^ cells.^135^ Together with arteriolar endothelial cells and stromal cells, are a source of extracellular matrix protein Del-1, a niche component, which promotes myelopoiesis from HSPCs.^137^

Aging induces vascular niche remodeling such as reduction of α-smooth muscle actin^+^ (α-SMA^+^) or NG2^+^ arterioles,^138^ but increased stromal populations.^71^ Moreover, nestin^+^ cells have enriched β_3_-adrenergic receptors of which stimulation reduces the retention signal CXCR12 resulting in HSC mobilization^123^ with circadian rhythm.^139^ This pathway is inhibited in mouse models of type 1 and type 2 diabetes (streptozotocin-induced and db/db mice, respectively).^140^ Exercise-increased HSPC quiescence is mediated by the reduction of leptin signaling on stromal cells.^141^

Endothelial cells are also sources of critical HSC maintenance factors, SCF^124^ (not as potent as LEPR^+^ stromal cells^131^) and CXCL12.^118^ Sinusoidal endothelial cell regeneration after irradiation is promoted by TNF-α released from granulocytes and subsequently supports hematopoietic regeneration.^49^ Endothelial cells are also a major source of Notch ligands DLL4 and DLL1. A loss of the vascular endothelial-expressed DLL4 (in VE-Cad-specific manner) skews BM hematopoiesis toward a significant transcriptional reprogramming and myeloid priming of HSPCs.^130^ Cellular aging of endothelial cells contributes to the aging of HSPCs as an infusion of young endothelial cells into aged mice is sufficient to reverse the myeloid bias of HSPCs.^142^ Aged BM endothelial cells have features, including high ROS, hypoxic, high IL-6, and low CXCL12 and DLL4.^142^ CD31 and endomucin identify sinusoids, arterioles, capillaries, and transition zone vessels, which connect arterioles with sinusoids near the bone (endosteum).^70^ In aged mice, endosteal vessels and transition zone vessels were reduced,^70^ suggesting the age-dependent niche remodeling. In mice with diet-induced diabetes, epithelial growth factor receptor (Egfr) signaling enriched in BM endothelial cells provides increased retention of HSPCs, and Egfr disruption in the endothelial cells causes HSPC proliferation, increased myelopoiesis, and impaired wound healing.^143^

Vascular permeability is implicated in acute neutrophil release^144^ and leukemic hematopoiesis.^145^ Less permeable arterial blood vessels maintain HSCs, whereas the more permeable sinusoids promote HSPC activation and are the exclusive site for immature and mature leukocyte trafficking to and from the BM.^146^ Acute IFN-α-mediated inflammation acts on HSC-niche endothelial cells, affecting vascularity and vessel leakage in the BM.^147^

#### Macrophages in the niche

Macrophages in the BM directly protect primitive HSPCs. These are identified as α-SMA-positive BM macrophages that are adjacent to HSPCs and release prostaglandin E2 limiting oxidative stress of HSPCs.^148^ As indirect regulation, other macrophages, BM mononuclear phagocytes marked by Gr-1^−^ CD115^int^ F4/80^+^ CD169^+^, maintain CXCR12 expression in nestin^+^ stromal cells regulating retention of HSPCs in the niche.^125^ This macrophage/stromal cell axis is inhibited when the macrophage engulfs aged or apoptotic neutrophils.^149^ BM macrophages facilitate hematopoietic tolerance and reduction of newly released myeloid cells in recurrent myocardial infarction, in which niche macrophages maintain HSPC through the adhesion molecule Vcam-1.^26^

Not only in the niche, but macrophages also produce/modulate niche factors in remote organs to regulate myelopoiesis in the BM. Phagocytosis of apoptotic neutrophils by dendritic cells and macrophages inhibits expression of IL-23 that in turn reduces G-CSF in plasma and granulopoiesis in the BM.^150,151^ In obese mice, TLR4-MyD88 signaling mediated IL-1β production from macrophage-containing adipose tissue.^61^ In aged mice, IL-1β is elevated in the BM along with increased caspase-1 activity and reduced efferocytosis in BM macrophages.^71^

#### Neutrophils in the niche

Neutrophils are the most abundant white blood cells in humans and undergo rapid turnover because of their short lifespan. Νeutrophils in various stages of their development and clearance regulate HSPCs.

In a normal steady state, some blood neutrophils return to the HSPC niche, especially when these neutrophils get senescent or aged rather than stimulated.^152^ In an experimental setting, BM-derived senescent neutrophils or blood-derived CD62L^lo^ neutrophils migrated to the vicinity of BM-resident macrophages, with 40% of neutrophils finding indirect contact with CD169^+^-resident macrophages and 78% within 20 μm.^149^ Thus, the majority of aged neutrophils are to be cleared by the macrophages when they migrate deep into BM.^153^ In contrast, nonaged or young neutrophils are not efficiently cleared by the resident macrophages in the BM despite the similar capacity of homing to the BM,^149^ suggesting that there is a significant number of neutrophils that home to the BM and subsequently recirculate in the blood.

These aged neutrophils homed to the BM negatively regulate the niche size for HSPCs according to circadian rhythm.^149^ Aged neutrophil uptake by macrophages reduces CXCL12 on CAR cells, which is essential in the adult BM to maintain the HSCs near the sinusoidal endothelium.^132^ Moreover, the modulation of the niche depends on macrophages and activation of cholesterol-sensing nuclear receptors.^149^ Since aging and clearance of neutrophils are controlled by circadian rhythm,^149,150^ neutrophils in the niche are essential for the circadian egress of HSPCs into the circulation in the normal steady state.

Both acute and chronic elevations in circulating neutrophils promote the release of HSPCs into blood, whereas their depletion promotes their retention in the BM.^149^ Moreover, in neutrophil-depleted mice, homing of MyP cells and LSK-HSPCs into the calvarial or femoral BM was increased by ∼40%.^149^

In acute inflammation caused by infection, neutrophils in the BM actively promote myelopoiesis of granulocyte progenitors^27,154^ and also promote granulocyte release by increasing permeability of BM sinusoid vessels.^144^ These actions of neutrophils are dependent on NOX2 NADPH oxidase. Similarly, in acute inflammation caused by ischemic tissue injury, NOX2 in neutrophils returning to the BM is required for reactive activation of HSPCs and myelopoiesis, whereas it simultaneously limits myelopoiesis at LSK levels.^29^ Since the homing of neutrophils to the BM, especially ones with aged phenotype, is increased by ischemic injury, neutrophils may be a key for emergency granulopoiesis and limiting hyperactivation of HSPCs, which collectively leads to resolution and tissue regeneration in the injured tissue.^29^ In myocardial infarction, danger proteins, S100A8/S100A9, released from neutrophils in the infarct tissue upregulates IL-1β levels in the blood, which in turn amplifies granulopoiesis and impairs tissue repair.^44^ In sepsis, lactate released from stimulated BM neutrophils not only increases vascular permeability but also induces G-CSF.^144^

In chronic inflammation with obesity, the S100A8/S100A9-IL-1β axis in adipose tissue promotes myelopoiesis.^155^ In hyperglycemia, neutrophil production and increased plasma levels of S100A8/S100A9 enhance myelopoiesis from MyP cells,^60^ indicating that stimulated neutrophils promote myelopoiesis in the BM. In addition, epinephrine stress promotes sustained neutrophil trafficking to the wound site through IL-6, resulting in impaired healing.^156^ Epinephrine treatment also causes a small population of the previously infused neutrophils to reenter the circulation.^152^ Therefore, prolonged circulation of stimulated neutrophils may promote myelopoiesis and myeloid accumulation.

BM neutrophils, specifically CXCR4^+^CXCR2^−^ preneutrophils, expressed HcrtR1 transcript encoding the receptor for hypocretin, which restricts M-CSF production by the preneutrophils. This neutrophil production of M-CSF is upregulated by sleep disturbance, promoting myeloid-biased hematopoiesis. Interestingly, hypocretin receptor-expressing preneutrophils reside near HSPCs, but sleep-induced hypocretin stimulation from the hypothalamus keeps restricting M-CSF (mature neutrophils have higher M-CSF expression),^157^ highlighting preneutrophils as niche cells for regulating myelopoiesis related to the severity of atherosclerosis. In aged mice, IL-1β is elevated in the BM, partly due to increased caspase-1 activity in BM neutrophils.^71^

As mentioned in the previous section, phagocytosis of apoptotic neutrophils by dendritic cells and macrophages inhibits expression of IL-23, a process that in turn reduces granulopoiesis by regulating production and blood levels of G-CSF.^150,151^ Further research will determine whether and how heterogeneous neutrophils (apoptotic, stimulated, preneutrophils, etc.) and their downstream processes provide beneficial or detrimental signals for myelopoiesis during wound healing.

#### T cells in the niche

Cytotoxic CD8^+^ T effector cells stimulate hematopoietic progenitors by promoting IL-6 release from BM MSCs through IFN-γ secreted by cytotoxic effector T cells. Adoptive transfer of these T cells to naive mice was sufficient to induce myeloid differentiation at the level of early multipotent hematopoietic progenitor cells.^37^ The BM is a known reservoir for regulatory T cells, and the specific depletion of these cells pointed to a role in controlling HSC quiescence and pool size.^134^

#### Megakaryocytes in the niche

Subsets of quiescent HSCs have been reported to associate with megakaryocytes.^78,126,158^ Platelet and myeloid-biased HSCs, marked by von Willebrand factor (vWF) expression, are highly enriched in megakaryocyte niches. Depletion of megakaryocytes selectively expands vWF^+^ HSCs, whereas the depletion of NG2^+^ arteriolar niche cells selectively reduces vWF^−^ lymphoid-biased HSCs.^134^ During normal/pathological aging, megakaryocyte numbers increase, but their interaction with quiescent HSC decreases because of increased physical distance between megakaryocytes and HSCs.^70^ Interestingly, β_3_-adrenergic receptor agonist can partially restore the interaction.^70^ Although dysregulated platelet functions are mediated by IL-6^159^ and IL-1β^160^ in diet-induced obesity of mice, megakaryocyte density in the BM is not affected by obesity^159^ or diabetes.^161^

#### Neurons in the niche

BM is extensively innervated by autonomic nerve fibers. The physiological roles of the sympathetic nervous system (SNS) have been shown to include regulation of hematopoiesis as a part of BM niche.^162^ Autonomic nerve denervation reduced the number of active TGF-β-producing cells and led to rapid loss of HSPCs from BM,^79^ and sympathetic nerve signals are critical for maintaining Schwann cells and nestin^+^ stromal cells.^163^ SNS-driven atherosclerosis led to a reduction in BM niches and extravasation of HSPCs out of the BM and into the spleen, suggesting severe BM dysfunction.^164^

Chronic socioenvironmental stress also increases the proliferation of HSPCs through upregulation of β_3_-adrenergic receptor signaling, leading to an increased output of neutrophils and inflammatory monocytes.^165^ However, another study showed that β_2_-adrenergic receptor promotes megakaryocyte differentiation, whereas β_3_-adrenergic receptor contributes to balance HSC lineage bias toward lymphoid production through Nos1-dependent nitric oxide production. In aged mice, β_2_/β_3_-adrenergic receptors exhibit opposite and niche-dependent regulation of myelopoiesis, where β_2_-adrenergic receptor signaling overriding β_3_-receptor increases generation of myeloid cells and platelets through an IL-6-dependent mechanism.^70^

### Hypoxia and oxidants in the niche

#### Hypoxia in the niche

Hypoxia governs important physiological and pathological processes in humans,^166^ where hypoxia-responsible cellular machinery such as hypoxia-inducible factor (HIF) are crucial. Prolyl hydroxylase in HIF is an oxygen-sensing element that regulates the stability of HIF protein.^167^ Recent advancements in technology allowed us to measure oxygen concentration in organs of live animals. The results from the *in vivo* oxygen measurement show that physiological oxygen concentration in most organs (5–8%) is much lower than the oxygen concentration in the ambient air (21%).^168^ Moreover, the HSC niche in the BM can have even lower oxygen (1.3%).^169^

Earlier studies, including ours, used staining with pimonidazole as a method to document low oxygen conditions of HSPCs.^170–172^ Pimonidazole hydrochloride, which acts as oxygen mimetic and irreversibly incorporates into cellular proteins only under hypoxic conditions, and the use of fluorescently labeled antibodies that recognize pimonidazole epitopes enables the indirect assessment of the intracellular hypoxic status.^173^ Ischemic injury^172^ and granulopoiesis stimulation by G-CSF^170^ increase pimonidazole staining in the BM with a pattern of the positive lesions (high signals in the area closed to the long bone surface). Thus, proliferation and activation of HSPCs in response to ischemic tissue injury or granulopoietic growth factor injection likely expand the hypoxia niche and induce intracellular hypoxia.

To measure hypoxia, popular approaches include the abovementioned pimonidazole hydrochloride, which can covalently bind to protein thiol groups under low O_2_ tension conditions, then analyzed by flow cytometry or immunofluorescence microscopy.^170–172,174–176^ Other chemicals, such as Ho 33342 dye, were used to detect low perfusion area as a surrogate for hypoxia.^171^ Quiescent or primitive HSPC has been shown to reside in the most hypoxic regions of the BM, where the Hif-1a expression was higher.^171,176^ *In vitro*, cocultured hematopoietic cells like to form a reservoir in the regions with low oxygen tension.^174^ More recently, a direct *in vivo* measurement of hypoxia in the BM of live mice has been achieved using two-photon phosphorescence lifetime microscopy, which utilized phosphorescent probe PtP-C34320 with a highly local O_2_ concentration-sensitive platinum porphyrin (PtP).^169^

Advances in imaging technologies using live imaging and the nanoprobe specifically reflective of ambient oxygen have challenged the concept of hypoxic niche in the BM, especially in the steady state. It showed that oxygen tension was lowest around sinusoids and highest near the endosteum (transplanted HSCs have been shown to home close to nestin^+^ cells, unexpectedly O_2_ tension is higher in nestin^+^ compared with nestin^−^ vessels).^169^ It is therefore likely that consumption of oxygen during hematopoiesis renders the marrow hypoxic, but that no distinct hypoxic region exists at the endosteum in the steady state. In line with this, the vast majority of long-term (LT)-HSCs are localized at the perivascular lesion.^116,129^ The entire BM space had much lower levels of oxygen compared with vessels entering the BM, however, this feature was largely lost when hematopoiesis was ablated by a cytotoxic drug or irradiation,^169^ considering that the vasculature was severely damaged. This highlights the importance of vascular barrier function to maintain hypoxia slope between BM space and arterioles.

Thus, previously detected hypoxia niche may be reflective of intracellular hypoxia or more likely metabolic state, rather than low oxygen in extracellular space around HSPCs. However, it is still possible that activation of HSPCs and/or active myelopoiesis may induce both intracellular and extracellular hypoxia. Interestingly, extracellular hypoxia is associated with increased vascular permeability in the BM, at least, in acute myeloid leukemia,^145^ suggesting that the function of BM endothelial cells may play a key role in environmental hypoxia in addition to consumption of oxygen during active hematopoiesis.

Animal study shows that when housed in a low O_2_ environment chronically, mouse HSPCs number increases, and BM cell engraftment is enhanced.^177^ Similarly, shielding mouse BM and human cord blood HSPC from ambient air exposure can help HSPC maintain quiescence, self-renewal, and increase transplantation efficacy.^178^ BM cells explanted from myelodysplastic syndrome patients, whose stem/progenitor cell functions are comprised, can be selected with severe hypoxia (0.1% O_2_) to a subset that is capable to escape hypoxia-induced apoptosis and endowed with stem cell potential, as determined by repopulation ability *in vitro*.^179^

When cocultured with MSCs *in vitro*, mouse HSPCs division is prolonged and migration is inhibited by hypoxia condition.^174^ Hypoxia can diminish the proliferation of *in vitro* cultured mouse HSPCs and accumulate more cells in G_0_, which maintains HSPCs in a more quiescent state.^180^ In human cord blood CD34^+^ cells, hypoxia induces similar cell cycle arrest and TGF-β treatment increases the percentage of quiescent cells.^181^ In young human subjects, intermittent hypoxia treatment causes HSPC count increase in peripheral blood, and total leukocyte, neutrophil, monocyte, and lymphocyte cell numbers are also upregulated.^182^

Technically, it is not easy to study HSPCs in physiological oxygen because their isolation involves multiple steps of enrichment and sorting traditionally set up in an atmospheric oxygen environment. Murine HSCs isolated in hypoxia are functionally better maintained in terms of repopulating capacity in serial transplantation.^178^ Also, hypoxia-inducible gene regulation^167,183,184^ and histone modification^185^ are very rapid (less than an hour) in other cell types. Future studies will determine whether the preparation of HSPCs in a hypoxia condition differs from that in the ambient oxygen. Moreover, at least in the steady state, tissue hypoxia and hypoxia-responsible elements have circadian oscillation.^167,183,184^ Whether this circadian hypoxia influences HSPC function needs to be investigated.

#### Oxidants in the niche

Oxidants are generated in intracellular and extracellular space as a result of the generation of ROS.^186^ Oxidants and ROS formed in extracellular space, transported through cellular membrane,^187^ influence cellular functions through redox signaling. The oxidative HSPC niche resulted from an accumulation of oxidants and ROS influences HSC functions in the short term and long term, as such oxidants regulate myeloid differentiation.^29,154^

ROS can be measured with various methods. ROS concentration in BM plasma could be measured by Amplex red, an H_2_O_2_-sensitive fluorescent probe.^27,154^ The 2′-7′-dichlorofluorescein diacetate can be used to measure ROS in viable cells.^188^ Dihydroethidium and isoluminol assay can be used to measure ROS in mice and isolated cells.^172^ CellROX dye staining was used to determine the ROS levels in cells.^29^ In addition, ROS species in the mitochondria of BM cells could be measured with MitoTracker orange CMTMRos or tetramethylrhodamine ethyl ester.^175,178^

ROS are generated by various cellular sources. The major sources of ROS are NADPH oxidase, mitochondria, uncoupled nitric oxide synthase, and xanthine oxidoreductase (XO).^189^ NADPH oxidases exist in different isoforms (NOX1, NOX2, NOX3, NOX4, and NOX5) and differ in their subunit organization and cellular expression.^190,191^ NADPH oxidase NOX2 converts oxygen to superoxide, which can be further converted to H_2_O_2_ and other ROS.^189^ NOX2 contains plasma membrane and cytosolic subunits, which assemble at the plasma membrane upon activating signals such as phosphorylation of its subunits.^189^

In the mitochondrial electron transport chain, complex I and complex III are the main sites of ROS generation.^189^ Mitochondria-derived ROS activates the NLRP3 inflammasome and production of inflammatory cytokines, such as IL-6 and TNF-α.^192^ ROS, such as superoxide, is also derived from XO, which is an enzyme that catalyzes the oxidation of hypoxanthine to xanthine and uric acid.^189^ Increased XO activity is observed in different inflammatory diseases such as airway inflammatory disorders, ischemia/reperfusion injury, atherosclerosis, diabetes, and autoimmune disorders such as rheumatoid arthritis.^193^

ROS and/or oxidant generation in the niche cells can activate signaling directly or indirectly through oxidation of lipids and/or protein. Ischemic tissue injury in mouse hindlimb increases both hypoxia and oxidants in the BM environment.^29,172^ A similar induction of oxidants in the BM was observed in infection-induced inflammation.^27,154^ In a hindlimb ischemia model, oxidized phospholipids, which can interfere with TLR-4 signaling, are increased in the BM, and this induction is NOX2 dependant.^29,172^ In these studies, NOX2 in neutrophils was identified as a source of oxidants.

Elevated ROS level in the BM caused by acute infection-induced inflammation or ischemic tissue injury can stimulate proliferation of MyP through a paracrine mechanism.^27,29^ Paradoxically, NOX2 knockout mice have persistent elevated LSK-HSPC activity and monopoiesis in both the steady state and after tissue injury, which is associated with decreased ROS and oxidant levels in the BM plasma.^29^ ROS derived from MSCs activates phosphoinositide 3-kinase (PI3K) in the signaling of phosphoinositide3,4,5-trisphosphate through oxidation of phosphatase and tensin homolog.^194^ This PI3K activation in BM stromal cells opens connexin channels to promote mitochondrial transfer to HSCs, promoting acute granulopoiesis in infections.^194^ Thus, ROS and oxidants in the niche promote early myelopoiesis mainly from differentiated progenitors but simultaneously limit hyperactivation of LSK cells.

It is important to note that intracellular ROS or oxidant generation in HSPCs, as opposed to their increased levels in the extracellular space, have different impacts on hematopoiesis and myelopoiesis as we discussed in the Signals for Chronic Activation of HSPCs section. Less permeable arterial blood vessels with higher environmental oxygen^169^ maintain HSCs in a low ROS state,^146^ whereas the more permeable sinusoids with lower environmental oxygen^146^ promote HSPC activation with higher ROS state.^146^ This suggests, similar to intracellular hypoxia, that intracellular ROS have implications in the metabolic state of HSPCs rather than ROS in the niche. Further researches will elucidate the link between the metabolic state and environmental oxidants.

### HSPC transfer in wound healing

Wound healing capacity of HSPCs has been studied in topical and systemic transfer settings as stem cell therapy. Along with rodent HSPCs, human CD34^+^ and/or CD133^+^ cells from adult BM, umbilical cords, or peripheral blood have exhibited their prohealing functions in animal models.^195–198^ CD34^+^ cells from these sources led to favorable outcomes in myocardial ischemia,^199,200^ critical limb ischemia,^201^ and chronic nonhealing ulcer.^202^ While CD34^+^ is enriched in HSPCs, it is not exclusive as the marker is also highly expressed endothelial lineage and subset of mesenchymal/epithelial lineages. Transfer of these cells have shown vasculogenic and/or immunomodulatory functions.^196,199,200^ However, high heterogeneity and a lack of expandability *ex vivo* are significant hurdles for broader clinical translation. Technological advancement in targeted selection of prohealing cells^197,198^ and in *ex vivo* expansion of HSCs^203^ might lower such hurdles in the future.

Other type of stem cells, such as MSCs and hair follicle stem cells, have played a bigger role in clinical application of stem cells to regenerative medicine and wound healing therapy. Compared with HSCs and HSPCs, these cells are scalable in autologous approach because they can be obtained from patient's excess adipose tissues, often as stromal vascular fraction. MSCs are also highly heterogenous, and their prohealing populations have been characterized based on immunomodulation and a secretion of prohealing properties, including growth factors/cytokines and EVs. The role of MSCs and other related therapies in wound healing is reviewed elsewhere and in other articles of this FORUM issue.

Wound healing pathway intersects with hair growth pathway as endogenous hair follicle stem cells give rise to skin epithelial cells in response to wounding^204^ in a particular hair growth cycle.^205^ Hair growth pathway can be stimulated by immune pathways.^206,207^ MSCs, which contain hair follicle stem cells^208^ and their conditioned media^209^ promote hair growth. Autologous growth factors, such as platelet-rich plasma, also promote hair growth and wound healing in human.^210^ However, the role of endogenous or exogenous HSCs in hair growth pathway remains unknown.

## Conclusion and Future Direction

We discussed the potential roles for HSPCs in wound healing responses with a focus on myelopoiesis. HSPCs participate in healing responses by quickly generating myeloid cells on demand, which in turn accumulate in the wound tissue. How quickly HSPCs generate myeloid cells determines the quality of acute inflammation (successful elimination of damaged tissues or infectious agents), which in turn leads to initiation of resolution and regenerative/healing processes.

**Figure 6.**
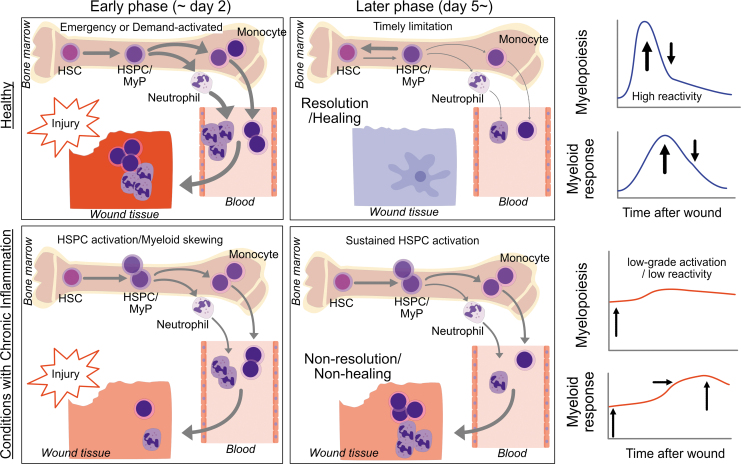
Myelopoiesis and HSPC activation in the healthy and in conditions with chronic inflammation. In the healthy, tissue injury can induce emergency or demand-activated myelopoiesis through activation of quiescent HSCs, resulting in reactive myelopoiesis and mobilization of effector immune cells such as neutrophils and inflammatory monocytes, which typically seen in the early phase (approximately day 2). This phase is followed by timely limitation of myelopoiesis and activated HSPCs, which in turn promotes resolution of inflammation and healing. In contrast, in conditions with chronic inflammation, HSPCs are activated in a basal condition (often seen as myeloid skewing with MyP expansion). However, these activated HSPCs lack reactivity and fail quickly enough to mobilize innate immune cells, leading to nonresolution and nonhealing with sustained HSPC activation. MyP, myeloid-committed progenitor.

Conditions with chronic inflammation—obesity, diabetes, hypercholesterolemia, atherosclerosis, aging, stress, and perhaps pre-existence of chronic wounds, are associated with immune dysregulation, and recent evidence highlight such dysregulations detected at an HSPC level through a cell-intrinsic and -extrinsic mechanisms. There are two features of HSPC dysfunction in conditions with chronic inflammation. The first is chronic activation and myeloid skewing. We see this as leukocytosis in the steady state, and leukocytosis is indeed recognized as a risk of additional conditions.^3–8^ The second feature is the slow or insufficient reactivity to additional insults such as wounds, injury, organ damage, and infection. Importantly, the low reactivity subsequently leads to prolonged activation of HSPCs, which in turn results in nonresolution and nonhealing (Fig. 6). Whether insufficient reactivity and prolonged activation of HSPCs is programmed in HSPCs will be a topic of discussion. Thus, epigenetic reprogramming in chronic conditions and the niche remodeling that can perpetuate the HSPC dysfunction are under investigation.

HSPC dysregulation is likely linked with the quality of generated myeloid cells in addition to the quantity generated from HSPCs. In this review, to focus on HSPCs, we discussed the increased quantity of myeloid cells through activated HSPCs. However, a recent conceptual update of innate immune memory includes the central trained immunity, where trained HSPCs give rise to monocyte-derived macrophages with enhanced effector functions (qualitatively).^92^ Moreover, clonal hematopoiesis, which is well documented in TET2 deficiency, will add another dimension of HSPC dysregulation—mutated HSPCs undergo clonal expansion that is associated with a risk of atherosclerosis.^105,211^ Defining disease- or condition-specific HSPCs that generate effector cells corresponding to certain diseases or conditions should enable us to develop more specific therapeutic targets with fewer side effects.^212^

As we saw the evolution in models of hematopoiesis (Fig. 1), technological advancements in single cell analysis and lineage tracing will help us to propose new models, including genetic/epigenetic regulation of HSPCs, niche remodeling, innate immune memory, and clonal hematopoiesis. Finally, although we focus on BM as an organ responsible for myelopoiesis, hematopoiesis from HSPCs takes place in the spleen, lymph, and other organs (HSPCs can migrate to peripheral organs).^213^

Introducing better healing in patients with chronic conditions may require the development of biomarkers that reflect a type of HSPC dysregulation. Besides, elucidating signals that limit activated HSPCs may be warranted. As we and others witnessed, a pathway inducing reactivity also timely limit myelopoietic activation of HSPCs.^27,29,154^ In line with this, exercise increases HSPC quiescence (limiting activity) but augment emergency hematopoiesis (increase reactivity).^141^

For translation into clinical setting, a cellular biomarker that reflects HSPC dysregulation in human conditions should be established. Leukocytosis has been proposed and identified as such biomarkers as we discussed. In addition, monocyte count^214,215^ and its subpopulation^216–221^ has been proposed to be a potential predictive or short-term marker of unfavorable outcomes in human. Blood monocyte subsets are mechanistically associated with the types (either inflammatory or prohealing) of wound macrophages in mouse models,^24,222^ however, such evidence in the human context is hard to get, and the relationship between BM HSPCs and wound macrophages is even harder to prove. Interaction of HSPCs with existing or developing drug may be studied in human.

The role of HSPCs in wound healing should be established by the efforts such as robust transcriptomic/epigenetic profiles in animals and humans, and finding key molecules and a development of HSPC-targeting drug. This research field is fertile and exciting, and we expect much exciting evidence that has clinical implications in near future.

## Summary

HSCs are responsible for the generation of effector innate immune cells, such as monocytes/macrophages and neutrophils. Dysfunction of HSCs is linked with risk factors for nonhealing wounds such as obesity, diabetes, aging, chronic infection, and atherosclerosis. These deteriorated health conditions are associated with low-grade chronic inflammation. It is also known that patients with nonhealing wounds or chronic wounds have a high risk of developing such conditions with chronic inflammation. Recent studies suggest that HSCs and HSPCs are a fundamental component of immune memory, which defines how many and what type of blood immune cells are produced in a timely manner. Around discoveries in HSC research, we may find a way to rejuvenate HSCs to enhance wound care and better protect organ damages.

**Take-Home Messages**

- HSCs can respond to wound injury and participate in healing processes.
- Dysfunction of HSCs is induced by many health conditions with chronic inflammation (*i.e.*, obesity, diabetes, and aging), which is associated with poor healing.
- Sufficient HSC response to wound injury is required for healing, while its prolonged activation may lead to poor healing with proinflammatory cell accumulation.
- HSCs and their progeny are regulated by signals from cytokines and growth factors, and their myelopoiesis can be modified by epigenetic programming.
- HSCs and their progeny are also regulated by their surrounding environment or niche, which include many types of cells, hypoxia, and oxidants.
- Understanding HSCs and hematopoiesis wound lead to biomarker discovery and establishment of novel therapies for better healing.

## About the Authors

**Norifumi Urao, MD/PhD,** is an Assistant Professor of Pharmacology at State University of New York Upstate Medical University. **Jinghua Liu, PhD,** is a Research Scientist, **Kentaro Takahashi, MD/PhD**, is a Postdoctoral Fellow, and **Gayathri Ganesh, MMS**, is a graduate student. The research group studies HSC dysfunction induced by metabolic syndrome and its negative impact on wound healing/tissue repair.

## Abbreviations and Acronyms

α-SMA: α-smooth muscle actin
AP-1: activator protein 1
BCG: Bacillus Calmette-Guérin
BM: bone marrow
CAR: CXCL12-abundant reticular
CMPs: common myeloid progenitors
CSF: colony-stimulating factor
CXCL12: C-X-C motif chemokine ligand 12
CXCR: C-X-C chemokine receptor
DAMPs: damage-associated molecular patterns
DLL: delta-like canonical Notch ligand
DNMTs: DNA methyltransferases
Egfr: epithelial growth factor receptor
EVs: extracellular vesicles
FoxO: forkhead box O
G-CSF: granulocyte colony-stimulating factor
Gfi1: growth factor independent 1 transcription repressor
GM-CSF: granulocyte-macrophage colony-stimulating factor
H3K27ac: histone 3 lysine 27 acetylation
H3K4me3: histone 3 lysine 4 trimethylation
HATs: histone acetyltransferases
HcrtR1: hypocretin receptor 1
HIF: hypoxia-inducible factor
HSCs: hematopoietic stem cells
HSPCs: hematopoietic stem progenitor cells
IFN: interferons
IL: interleukin
IRF: interferon regulatory factor
JAK: Janus kinases
JNK: c-Jun N-terminal kinase
KDM5: histone demethylase 5
Ldlr: low-density lipoprotein receptor
Lepr: leptin receptor
lncRNA: long noncoding RNA
LPS: lipopolysaccharide
LSK: Lineage^−^Sca1^+^cKit^+^
MALAT1: metastasis-associated lung adenocarcinoma transcript 1
MAPK: mitogen-activated protein kinase
M-CSF: macrophage colony-stimulating factor
MPPs: multipotent progenitors
MSC: mesenchymal stem (stromal) cell
MyP: myeloid progenitors
Nes: Nestin
NF-κB: nuclear factor kappa-light-chain-enhancer of activated B cells
NG2: neural/glial antigen 2
NLRP3: NOD^−^, LRR^−^, and pyrin domain-containing protein 3
NOX: NADPH oxidase
PAMPs: pathogen-associated molecular patterns
PI3K: phosphoinositide 3-kinase
PtP: platinum porphyrin
ROS: reactive oxygen species
SCF: stem cell factor
SLAM: signaling lymphocytic activation molecule
SNS: sympathetic nervous system
Src: proto-oncogene tyrosine-protein kinase Src
STAT: signal transducer and activator of transcription protein
TDG: thymine DNA glycosylase
TET: ten-eleven translocation
TGF-β: transforming growth factor-beta
TLRs: toll-like receptors
TNF: tumor-necrosis factor
TRIF: TIR-domain-containing adapter-inducing interferon-β
VE: vascular endothelial
vWF: von Willebrand factor
XO: xanthine oxidoreductase
